# Target-selective homologous recombination cloning for high-throughput generation of monoclonal antibodies from single plasma cells

**DOI:** 10.1186/1472-6750-11-39

**Published:** 2011-04-13

**Authors:** Nobuyuki Kurosawa, Megumi Yoshioka, Masaharu Isobe

**Affiliations:** 1 Laboratory of Molecular and Cellular Biology, Faculty of Science and Engineering, Graduate School, University of Toyama, 3190 Gofuku, Toyama-shi, Toyama, 930-8555, Japan; 2 Graduate School of Innovative Life Science, University of Toyama, Toyama-shi, Toyama, 930-8555, Japan; 3 Laboratory of Molecular and Cellular Biology, Faculty of Science and Engineering, Graduate School, University of Toyama, 3190 Gofuku, Toyama-shi, Toyama, 930-8555, Japan

## Abstract

**Background:**

Molecular cloning of functional immunoglobulin genes from single plasma cells is one of the most promising technologies for the rapid development of monoclonal antibody drugs. However, the proper insertion of PCR-amplified immunoglobulin genes into expression vectors remains an obstacle to the high-throughput production of recombinant monoclonal antibodies.

**Results:**

We developed a single-step cloning method, target-selective homologous recombination (TS-HR), in which PCR-amplified immunoglobulin variable genes were selectively inserted into vectors, even in the presence of nonspecifically amplified DNA. TS-HR utilizes Red/ET-mediated homologous recombination with a target-selective vector (TS-vector) with unique homology arms on its termini. Using TS-HR, immunoglobulin variable genes were cloned directly into expression vectors by co-transforming unpurified PCR products and the TS-vector into *E. coli*. Furthermore, the high cloning specificity of TS-HR allowed plasmids to be extracted from pools of transformed bacteria without screening single colonies for correct clones. We present a one-week protocol for the production of recombinant mouse monoclonal antibodies from large numbers of single plasma cells.

**Conclusion:**

The time requirements and limitations of traditional cloning procedures for the production of recombinant immunoglobulins have been significantly reduced with the development of the TS-HR cloning technique.

## Background

Molecular cloning of immunoglobulin variable (V) genes from single isolated plasma cells is a powerful tool for the unbiased generation of recombinant monoclonal antibodies [1-3]. Fluorescence-activated single-cell sorting followed by a single-cell reverse-transcription polymerase chain reaction (RT-PCR) has been shown to enable the high-throughput production of V gene DNA fragments [4-6]. However, the proper insertion of the V gene DNA fragments into expression vectors remains an obstacle to the high-throughput production of recombinant monoclonal antibodies.

The most commonly employed cloning method for introducing V gene DNA fragments into vectors is ligation-dependent cloning [1,3,5,6]. However, ligation-dependent cloning is often hampered by the requirement for multiple rounds of enzyme treatments and purification of both the inserts and vectors. Furthermore, the limited number of appropriate restriction enzyme sites in the insert and vector DNA limits flexibility in constructing recombinant molecules. Recently, site-specific recombination-based cloning has emerged as a ligation-independent cloning method [7-9]. However, this technology introduces extra codons into the gene's primary sequence at the site of recombination, which may interfere with the folding and stability of the resulting protein. In contrast, homologous recombination technologies enable the seamless insertion of any DNA fragment at any desired position [10]. Recently, In-Fusion homologous recombination, which can join a DNA fragment and a linear vector with 15 bases of homology at their ends, was used in the production of recombinant monoclonal antibodies [11]. Although the In-Fusion technology offers several advantages as a high-throughput procedure, the amplified V gene DNA fragments must be purified to remove salts, primers and nonspecifically amplified DNA before the reaction. Subsequent steps are also necessary to screen single colonies for a correct clone, which can result in additional labor and increased cost. Thus, a method for the high-throughput cloning of PCR-amplified V gene DNA fragments into vectors that bypasses these tedious preliminary steps is needed.

**Figure 1:**
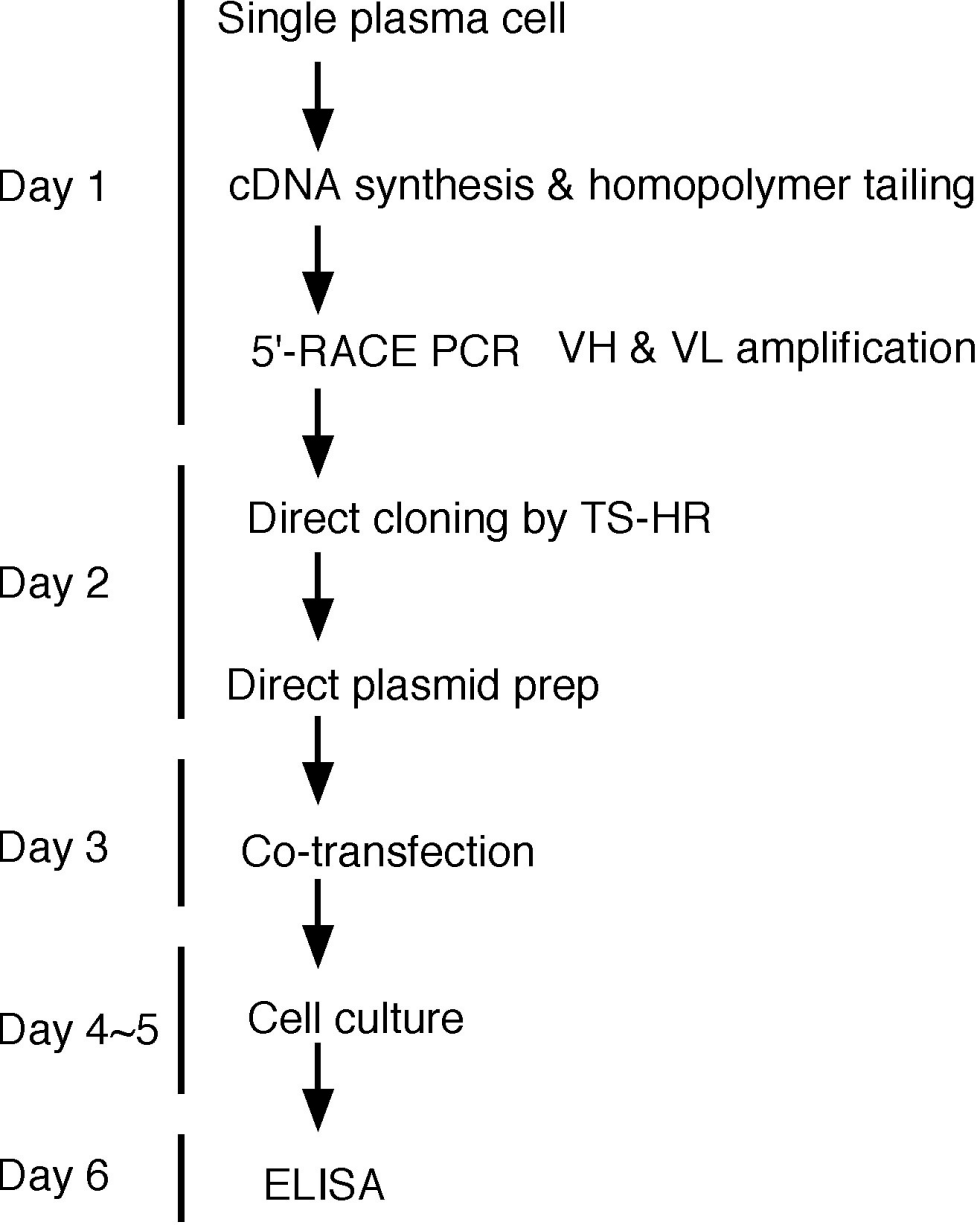
**Flow chart summarizing the production of recombinant antibodies from single plasma cells**. 3' Homopolymer-tailed cDNA was synthesized from single plasma cells by droplet-based solid-phase cDNA synthesis. The VH and VL genes were amplified by two rounds of PCR. The unpurified VH and VL genes were directly inserted into the corresponding vectors by TS-HR. After the transformation, the bacteria were directly cultured in liquid medium, and pools of expression plasmids were extracted without screening single colonies for correct clones. The cognate pairs of the expression plasmids were co-transfected into 293FT cells. Two days after the transfection, the antibodies secreted into the cell culture medium were tested for reactivity by ELISA.

We have developed a method, termed target-selective homologous recombination (TS-HR), in which PCR-amplified V gene DNA fragments can be selectively cloned into vectors, even in the presence of nonspecifically amplified PCR products. This system, together with the additional methods described herein, circumvents the problems associated with the amplification and cloning of V gene DNA fragments and provides a system for the high-throughput production of recombinant mouse monoclonal antibodies from large numbers of single plasma cells within a one-week time span (Figure 1).

## Results and Discussion

### Low cloning specificity in conventional, Red/ET-mediated homologous recombination

**Figure 2:**
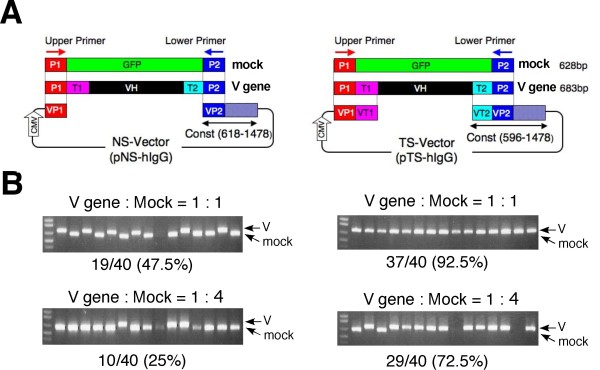
**Pilot experiment demonstrating the cloning specificity of the TS-HR technique**. (A) Schematic representation of the homologous regions between the vectors and the DNA fragments. (B) Result of the conventional homologous recombination (left) and the TS-HR method (right). The PCR-amplified V gene sequence and mock DNA were mixed in the indicated molar ratios and introduced into *E. coli *with either non-selective (pNS-hIgG) or selective vector (pTS-hIgG). Colony PCR was performed, and the amplified DNA fragments were separated by agarose gel electrophoresis. Forty colonies were analyzed, and data from a representative experiment are presented; the cloning specificity is expressed as a percentage of the total number of colonies analyzed.

Red/ET-mediated recombination is a powerful homologous recombination system based on the function of either the Red operon of lambda phage or RecE/RecT from Rac phage [12-15]. To evaluate the cloning specificity of conventional Red/ET-mediated homologous recombination, we conducted a pilot experiment using a linear nonselective vector (NS-vector) and an artificially amplified V gene and mock DNA. The V gene was composed of an upper primer-derived sequence (P1), a poly-dG/dC sequence (T1), a human VH sequence, part of the constant gene sequence (T2) and a lower primer-derived sequence (P2). The mock DNA was composed of the P1 sequence, part of the GFP sequence and the P2 sequence. The linear NS-vector contained homology arms at its ends (VP1 and VP2) that were homologous to the P1 and P2 sequences, respectively (Figure 2A, left). When the V gene and the mock DNA were mixed in a 1:1 molar ratio and introduced into competent cells with the NS-vector, each DNA fragment was inserted into the NS-vector with the same probability (Figure 2B, upper left). Transformation of the bacteria with the V gene and the mock DNA fragment in a 1:4 molar ratio resulted in the insertion of the V gene in 25% of the clones (10 out of 40 clones) (Figure 2B, lower left). Because the conventional homologous recombination reaction was mediated through the primer-derived sequences, the DNA fragments containing the primer sequences at their ends were non-selectively inserted into the NS vector.

### High cloning specificity of TS-HR

To selectively clone the V gene, even in the presence of nonspecific amplified DNA, we constructed a new vector: the target-selective vector (TS-vector). The TS-vector has homology arms (VP1-VT1 and VP2-VT2) on its ends. The VP1 (VP2) sequence shares homology with the P1 (P2) sequence, which is a primer-derived sequence. VT1 (VT2) shares homology with T1 (T2), which is a part of the V gene-specific sequence internal to P1 (P2) (Figure 2A, right). When the V gene and the mock DNA were mixed in a 1:1 molar ratio and introduced into competent cells with the linear TS-vector, 92.5% of the analyzed colonies contained the V gene (Figure 2B, upper right). Transformation of the bacteria with the V gene and the mock DNA in a 1:4 molar ratio resulted in the insertion of the V gene in 72.5% of the analyzed colonies (29 out of 40 clones) (Figure 2B, lower right). This result confirms the high cloning specificity of the TS-HR method, even in the presence of an excess amount of nonspecific amplified DNA.

**Figure 3:**
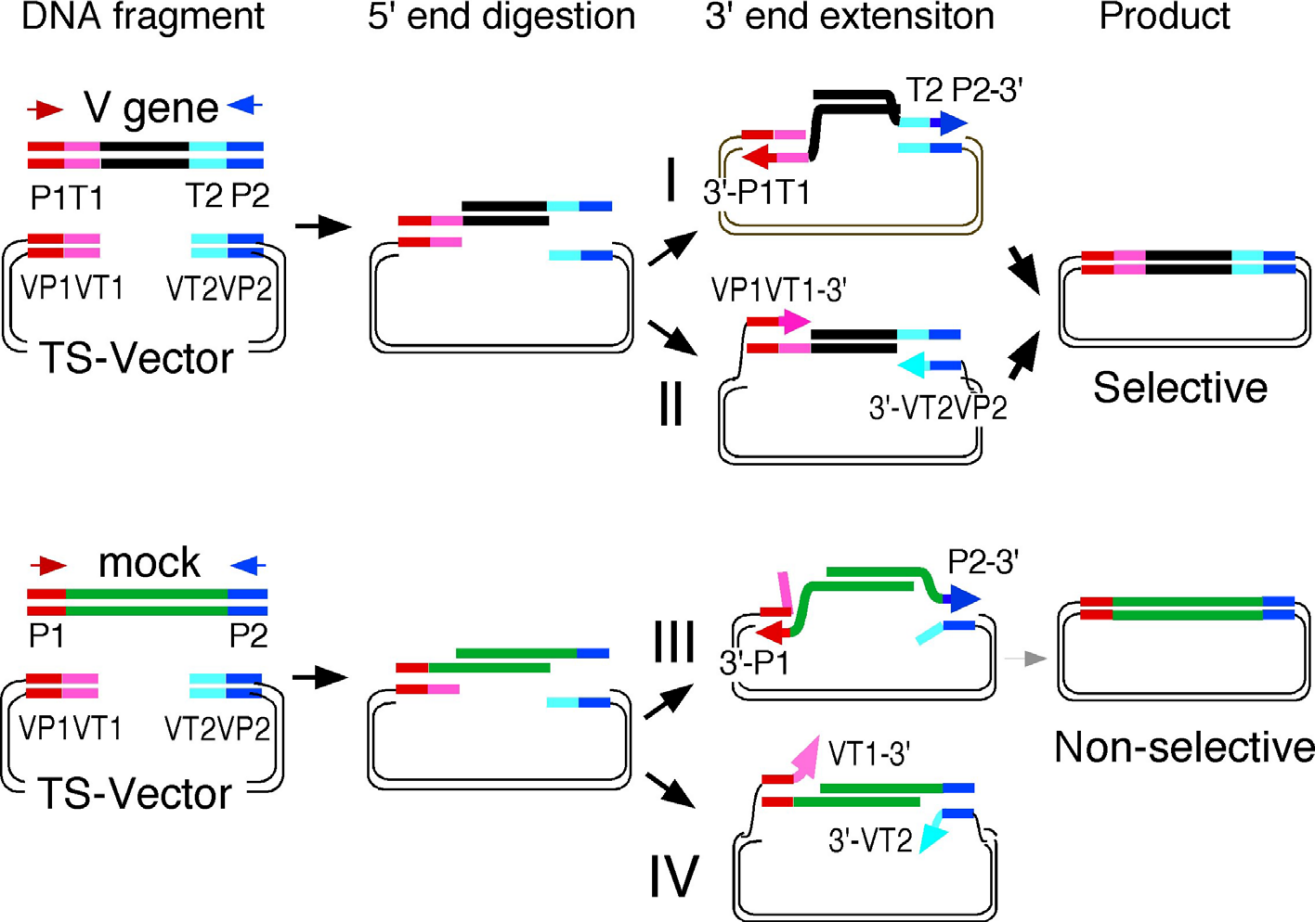
**Schematic illustration of TS-HR**. In *E. coli*, linear DNA was digested by the Redα 5'→3' exonuclease, resulting in the generation of 3' single-stranded tails. Homologous recombination of the V gene strands and the TS-vector, which is mediated by the long overlapping sequences (~50 bp), proceeds by 3' end extension from P1 and P2 (route I). However, homologous recombination of the mock DNA strands and the TS-vector is inefficient because of their short overlapping sequences (route III). Homologous recombination of the TS-vector and the V gene, which is mediated through the long overlapping sequences (~50 bp), proceeds by 3' end extension from VT1 and VT2 (route II). However, homologous recombination of the TS vector and the mock DNA does not occur because strand extension from VT1 and VT2 is blocked by 3' end mismatches (route IV).

The mechanism for selective cloning in the TS-vector is illustrated in Figure 3. The 5' ends of the linear DNA are removed by the Redα 5'→3' exonuclease in *E. coli*, resulting in the generation of 3' single-stranded tails from the TS-vector, target and non-target DNA. When the single-stranded tails of the target DNA anneal to the complementary single-stranded tails of the TS-vector through the long homology overlaps (P1-T1 to VP1-VT1 and P2-T2 to VP2-VT2), the recombination reaction proceeds via route I. When the single-stranded tails of the mock DNA anneal to the complementary single-stranded tails of the TS-vector through the short homology overlaps (P1 to VP1 and P2 to VP2), the recombination reaction proceeds via route III. However, the route I reaction dominates over the route III reaction because the homologous recombination reaction depends on the length of the homology overlap. The homologous recombination reaction also proceeds when the single-stranded tails of the TS-vector anneal to the complementary single-stranded tails of the target DNA (Figure 3, route II). However, the homologous recombination reaction does not proceed when the single-stranded tails of the TS-vector anneal to the single-stranded tails of the mock DNA. The 3' ends of the single-stranded tails of the TS vector (VT1 and VT2) fail to serve as primers for strand extension due to the 3' end mismatches (Figure 3, route IV).

### Application of TS-HR for high-throughput production of recombinant mouse antibodies

**Figure 4:**
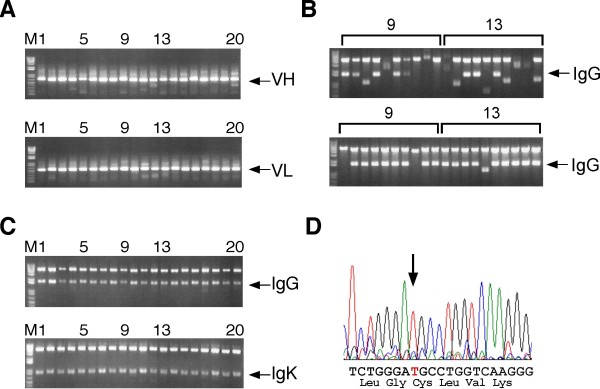
**High-throughput production of recombinant mouse monoclonal antibodies by the TS-HR technique**. (A) Amplification of cognate pairs of VH and VL genes from single plasma cells by 5' RACE PCR. (B) The PCR product obtained (A, upper, lane 9 and 13) was directly inserted into pNS-mIgG by conventional homologous recombination (upper) or into pTS-mIgG by TS-HR (lower). After the transformation, bacteria were plated on agar plates containing 1% sucrose and kanamycin. Ten colonies were cultured, and plasmids were extracted from the overnight culture. Plasmids digested with *Sal*I/*Not*I were electrophoresed in a 1% agarose gel. (C) The amplified VH and VL genes obtained in (A) were directly cloned into linear pTS-mIgG and pTS-mIgK, respectively, by TS-HR. After the transformation, bacteria were directly inoculated into LB medium containing 1% sucrose and 50 μg/ml of kanamycin, and pools of plasmids were prepared from the overnight culture. Plasmids digested with *Sal*I/*Not*I were electrophoresed in a 1% agarose gel. (D) DNA sequencing of the plasmid obtained in (C) confirmed the in-frame insertion of the V gene into the plasmid. A representative sequence of data from the plasmid originating from (C, upper) lane 9 is shown; the arrow indicates the DNA fragment-vector junction of the Ig constant gene.

To demonstrate the usefulness of TS-HR, we amplified the variable region of the immunoglobulin heavy chain (VH) and the variable region of the immunoglobulin light chain (VL) genes from single mouse plasma cells by 5' RACE PCR and attempted to insert the fragments into the expression vectors. The pairs of VH and VL genes were successfully amplified from single plasma cells (Figure 4A). When conventional Red/ET-mediated homologous recombination was conducted with a non-selective vector (pNS-mIgG) and unpurified PCR products from lane 9 or 13, only 50% and 30% of the colonies contained the VH gene were obtained, respectively (Figure 4B, upper). These results clearly indicate that purification of the V gene is required prior to the conventional homologous recombination reaction. However, when TS-HR was conducted with a V gene-selective vector (pTS-mIgG) and unpurified PCR products from lane 9 or 13, 80% and 90% of the colonies contained the VH gene were obtained, respectively (Figure 4B, lower). Theses results were in good agreement with the pilot experiment in Figure 2.

In light of the high cloning specificity of the TS-HR technique, we prepared the immunoglobulin expression plasmids without screening single colonies for a correct clone. When the pool of plasmids was extracted from the bacteria directly cultured in liquid medium after transformation, all 40 examined plasmids contained an insert of the expected size (Figure 4C). Furthermore, the V gene DNA sequence was present in all of the plasmids examined, and the cloning reaction proceeded as expected, with no insertions or deletions at the cloning junctions (Figure 4D and Table 1). We also observed the successful in-frame insertion of VH genes belonging to G1, G2a and G2b, indicating the unbiased insertion of the IgG subclass (Table 1).

**Table 1:** V-(D)-J repertoire of cloned V genes.

	Gamma Chain				Kappa Chain	
	
	V	D	J	isotype	V	J
1	IGHV10-1*01	IGHD2-4*01	IGHJ3*01	G2b	IGKV6-32*01	IGKJ5*01
2	IGHV1-5*01	IGHD2-4*01	IGHJ1*03	G2a	IGKV1-117*01	IGKJ4*02
3	IGHV1-4*01	IGHD1-1*02	IGHJ1*03	G1	IGKV6-15*01	IGKJ5*01
4	IGHV1-26*01	IGHD4-1*02	IGHJ2*01	G2b	IGKV6-17*01	IGKJ1*01
5	IGHV5-17*01	IGHD2-3*01	IGHJ3*01	G2b	IGKV5-43*01	IGKJ5*01
6	IGHV4-1*01	IGHD3-2*01	IGHJ2*01	G1	IGKV6-23*01	IGKJ4*01
7	IGHV1-22*01	IGHD2-12*01	IGHJ3*01	G1	IGKV14-111*01	IGKJ4*01
8	IGHV1-26*01	IGHD2-2*01	IGHJ3*01	G1	IGKV4-78*01	IGKJ1*01
9	IGHV5-17*01	IGHD2-3*01	IGHJ3*01	G2b	IGKV5-43*01	IGKJ5*01
10	IGHV5-16*01	IGHD3-2*01	IGHJ2*01	G1	IGKV6-20*01	IGKJ4*02
11	IGHV1-19*01	IGHD4-1*01	IGHJ2*01(a)	G1	IGKV1-135*01	IGKJ2*03
12	IGHV1-9*01	Not identified	IGHJ4*01	G2b	IGKV1-99*01	IGKJ1*01
13	IGHV1-62-2*01	IGHD1-1*01	IGHJ2*01(a)	G2a	IGKV12-46*01	IGKJ1*01
14	IGHV1-70*01(p)	IGHD3-1*01	IGHJ4*01	G1	IGKV1-117*01	IGKJ2*01
15	IGHV8-12*01	IGHD1-1*01	IGHJ1*01	G1	IGKV10-96*01	IGKJ1*01
16	IGHV5-4*01	IGHD4-1*01	IGHJ1*03	G1	IGKV10-94*03/08	IGKJ1*01
17	IGHV1-53*01	IGHD1-1*01	IGHJ2*01	G2b	IGKV12-41*01	IGKJ1*01
18	IGHV1-22*01	IGHD2-12*01	IGHJ3*01	G1	IGKV14-111*01	IGKJ4*01 or 02
19	IGHV1-66*01	IGHD1-1*01	IGHJ2*01(a)	G1	IGKV5-45*01	IGKJ2*03
20	IGHV5-16*01	IGHD3-3*01	IGHJ1*03	G2b	IGKV1-115*01(p)	IGKJ5*01

Pools of plasmids originating as described in Figure 4C were sequenced, and their V-(D)-J-(H) repertoires were determined.

**Figure 5:**
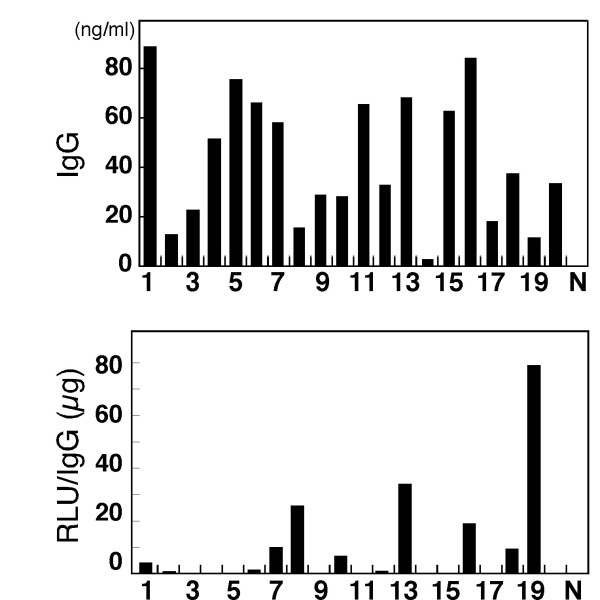
**Expression of recombinant mouse antibodies**. (A) The concentration of the recombinant antibodies in the supernatant of cultured 293FT cells was determined by sandwich ELISA. N indicates a negative control; all of the incubation steps were identical, except that non-transfected cell culture medium was used. (B) The specific activity of the recombinant antibodies was expressed as RLU/s/IgG (μg).

Co-transfection of the cognate pair of heavy chain and light chain expression plasmids into 293FT cells resulted in the secretion of recombinant antibodies (19 out of 20); seven clones specifically reacted with the antigen (Figure 5).

The major advantages of the TS-HR system include its specificity, rapidity and robustness. By using the TS-HR technique, the time-consuming processes inherent in current cloning methods, such as the purification of the V gene DNA fragment and the plating of transformed bacteria to screen single colonies for correct clones, are dramatically reduced. Due to the simplicity of our system, dozens of expression plasmids can be produced simultaneously, enabling us to produce recombinant antibodies from large numbers of single plasma cells. In addition, unlike other cloning systems, Red/ET-mediated recombination can be performed inexpensively in large quantities, saving investigators significant time and expense. The TS-HR system can be applied to the selective cloning of DNA fragments in which primer regions and their internal gene-specific sequences are constant but the intervening regions vary; T-cell receptor genes are an example of such a target sequence.

## Conclusion

The method described here overcomes several major obstacles to the cloning of PCR-amplified V gene DNA fragments into expression vectors. First, unlike currently employed cloning techniques, the TS-HR method does not require purification of the V gene. Second, TS-HR does not require the screening of transformed bacterial colonies for correct clones. These features suggest that TS-HR is directly applicable to high-throughput applications.

## Methods

### Materials

The animal experiments in this study were approved by the Committee on Animal Experimentation at the University of Toyama. Seven-week-old female ICR mice were immunized twice (at intervals of one month) intradermally in the footpad with 50 μl of a 50:50 water-in-oil emulsion containing 25 μg of egg albumin (Nakarai Tesque). Two to four weeks after the second immunization, popliteal lymph nodes were surgically removed, and plasma cells were isolated using the CD138+ plasma cell isolation kit according to the manufacturer's instructions (Miltenyi Biotec). The isolated plasma cells were microscopically identified after staining with an anti-CD138 antibody. Single cells were captured with a standard micromanipulator mounted on an inverted microscope and suspended in 3 μl of a lysis/binding solution (100 mM Tris HCl pH 7.5, 500 mM LiCl, 1% lithium dodecyl sulfate and 5 mM dithiothreitol) containing 3 μg of oligo-dT magnetic beads.

### Synthesis of 3' homopolymer-tailed cDNA from single cells

3' Homopolymer-tailed cDNA from single plasma cells was prepared by a droplet-based solid-phase cDNA synthesis termed Magnetic-beads Reaction through Arrayed Hanging Drops (MAGrahd), which is described in more detail below. Briefly, 3 μl each of single-cell lysate containing oligo-dT magnetic beads, washing solution 1 (10 mM Tris HCl pH 7.5, 150 mM LiCl, 0.1% lithium dodecyl sulfate and 1 mM EDTA), washing solution 2 (75 mM KCl, 3 mM MgCl_2_, 0.1% Triton X-100, 5 mM DTT and 2 units of RNase inhibitor), reverse transcription solution (washing solution 2 containing 10 units of SuperScript III reverse transcriptase), washing solution 3 (50 mM potassium phosphate pH 7.0, 0.1% Triton X-100 and 4 mM MgCl_2_), terminal transferase solution (washing solution 3 containing 10 units of terminal deoxynucleotidyl transferase), washing solution 4 (TE buffer containing 0.1% Triton X-100) and PCR solution (1x ExTaq buffer containing 0.1 mM dNTPs and 0.1% Triton X-100) were deposited on the surface of a hydrophobic film to form a droplet.

The mRNA immobilized on the magnetic beads was extracted from the cell lysis droplet with an externally applied magnetic force and sequentially transferred into droplets of washing solution 1, washing solution 2 and the reverse transcription solution. After 15 minutes of reverse transcription at 37°C, the cDNA immobilized on the magnetic beads was sequentially transferred into a droplet of washing solution 3 and a droplet of terminal transferase solution for 3' poly-dG tailing. After the terminal transferase reaction had proceeded for 15 minutes at 37°C, the homopolymer-tailed cDNA immobilized on the magnetic beads was sequentially transferred into droplets of washing solution 4 and PCR solution.

### Amplification of VH and VL genes from single plasma cells

DNA fragments of the VH and VL chain genes from single mouse plasma cells were amplified by the rapid amplification of the 5' cDNA ends by polymerase chain reaction (5' RACE-PCR) [16]. Briefly, the first round of PCR was performed with a forward primer (AP3dC-S) specific for the homopolymer-tail and reverse primers specific for the respective immunoglobulins' gamma and kappa constant regions (mIgG1st-AS and mIgK1st-AS). The second round of PCR was performed with a forward primer (MCSAP3-S) that annealed to the first PCR forward primer and with the respective nested reverse primer specific for the IgG (mIgG2nd-AS) or IgK (mIgK2nd-AS) constant regions. The PCR primers used for the amplification of the mouse VH and VL genes are detailed in Table 2. PCR was performed using the ExTaq DNA polymerase in a BIO-RAD MyCycler (35 cycles of reaction at 94°C for 30 seconds, 68°C for 40 seconds and a final extension at 72°C for 3 minutes).

**Table 2:** Primers used for amplifying the mouse VH and VL genes.

Use	Primer	Sequence
1st PCR upper	AP3dC-S	CGGTACCGCGGGCCCGGGATCCCCCCCCCCCCCDN

2nd PCR upper	MCSAP3-S	CTTCGAATTCTGCAGTCGACGGTACCGCGGGCCCGGGA

IgG 1st PCR lower	mIgG1st-AS	ACCYTGCATTTGAACTCCTTGCC

IgG 2nd PCR lower	mIgG2nd-AS	CTGGACAGGGATCCAGAGTTCCA

IgK 1st PCR lower	mIgK1st-AS	ACTGCCATCAATCTTCCACTTGACA

IgK 2nd PCR lower	mIgK2nd-AS	ACTGAGGCACCTCCAGATGTTAACT

### Plasmid construction

A human IgG constant region was amplified by RT-PCR with human B cell cDNA as a template. An 882-bp DNA fragment encoding the human IgG constant gene (596-1478 of the nucleotide accession number AK301389) was amplified by PCR with the primer 596/617-S (5'-- GCCCGGGATCCGATATCACGTGGAACTCAGGCGCCCTGACC--3', restriction enzyme sequences underlined) and 1478-AS (5'--GAGTCGCGGCCGCCGTCGCACTCATTTACCCGGAG--3'). The amplified gene was digested with *BamH*I and *Not*I and subcloned into the *Bam*HI/*Not*I sites of the pDsRed2-N1 vector (Clontech) to construct phIgG-C. The *sacB *gene was amplified from the pDNR1 vector (Clontech) by PCR with the primers 5'--CCCGATATCGATCCGACGTCCACATATACC-3' and 5'-CACGTGATATCGGCATTTTCTTTTGC--3', digested with *Eco*RV and inserted into the *Eco*RV site of phIgG-C to construct pIn-hIgG. The dC13/dG13 linker DNA was generated by annealing the oligonucleotides 5'--GATCCCCCCCCCCCCCGATATC-3' and 5'--GATCGATATCGGGGGGGGGGGG -3' and inserting the fragment into the *Bam*HI site of phIgG-C to produce the V gene-selective vector pTS-hIgG. A non-selective vector, pNS-hIgG, was amplified from pTS-hIgG by PCR with the primers 618/642-S (5'-- CAGCGGCGTGCACACCTTCCCGGCT--3') and AP3MCS-AS (5'--TCCCGGGCCCGCGGTACCGTCGACTGCAGAATTCGAAG--3').

The mock DNA fragment and the V gene DNA fragment used for the pilot experiments were amplified by PCR using plasmids as templates; the primers used were an upper primer, MCSAP3-S and a lower primer, 618/642-AS (5'-- AGCCGGGAAGGTGTGCACGCCGCTG --3'). The mock fragment (628 bp) consisted of the upper primer-derived sequence (P1 region), part of the GFP gene sequence (non-target DNA) and the lower primer-derived sequence (P2 region). The target DNA fragment (683 bp) consisted of the upper primer-derived sequence (P1 region), the poly-dC/dG sequence (T1 region), the human immunoglobulin VH region, part of the human immunoglobulin gamma heavy chain constant region that included nucleotides 596-617 (T2 region) and the lower primer-derived sequence (P2 region). The amplified DNA fragments were purified by S-400 spin column. A schematic illustration of the DNA fragment and the vectors is shown in Figure 2A.

A DNA fragment encoding 901 bp of the mouse IgG constant region (559-1460 of nucleotide accession number AB097849) was amplified with the primers 5'-- GATATCACGTGTGCCTGGTCAAGGGCTATTTCCCTGAG-3' (restriction site underlined) and 5'-- CTCCGCGGCCGCTGGGATCATTTACCAGGAGAGT-3'. A DNA fragment encoding 309 bp of the mouse IgK constant region (441-750 of nucleotide accession number AF466770) was amplified with the primers 5'--GATATCACGTGCTGTATCCATCTTCCCACCATCC-3' and 5'--TCTCGCGGCCGCTGTCTCTAACACTCATTCCTG-3'. The pTS-mIgG and pTS-mIgK plasmids were constructed by replacing the human IgG constant gene of pTS-hIgG with the PmlI/NotI-digested mouse IgG or IgK constant region, respectively. A linear pNS-mIgG, a non-selective vector, was amplified from pTS-mIgG by PCR with the primers MCSAP3-AS and mIgG2nd-S (5'-- TGCCTGGTCAAGGGCTATTTCCCTGAG-3').

### Red/ET-mediated recombination

Red/ET-mediated recombination was carried out as described previously [15]. Vectors were linearized outside of the homology region by digestion with *Eco*RV. The linear vector (0.1 μg) and the PCR-amplified DNA were mixed in a 1:2 molar ratio and transformed into competent cells. After recombination, bacteria were plated on agar plates containing 1% sucrose and 50 μg/ml kanamycin or directly inoculated into LB medium containing 1% sucrose and 50 μg/ml kanamycin.

### Colony PCR

Bacterial colonies were suspended in 100 μl of PBS-0.1% Triton X-100 solution and heated at 95°C for 5 minutes to extract the plasmid DNA. For PCR, 10 pmol of primers (MCSAP3-S and 618/642-AS) was added to 1 μl of the above cell-body heat-extracted solution, and PCR was performed in 50 μl of a reaction system using the ExTaq DNA polymerase (30 cycles of reaction at 94°C for 30 seconds and 68°C for 40 seconds).

### Sequence analysis

Nucleotide sequences were determined with an Applied Biosystems 373 DNA sequencer using a BigDye Terminator v.3.1 Cycle Sequence Kit (Applied Biosystems). The V-(D)-J sequences were aligned with IMGT/V-QUEST (http://imgt.cines.fr/IMGT_vquest/share/textes/)[17].

### Expression of recombinant mouse antibodies

5' RACE-amplified mouse VH and VL genes were cloned in-frame into pTS-mIgG and pTS-mIgK using TS-HR methodology. Plasmids were purified with PureYield Plasmid Miniprep kits (Promega). Cognate pairs of VH and VL genes were co-transfected into 293FT cells grown in 24-well culture dishes with FuGENE HD Transfection Reagent (Roche). Two days after transfection, cell culture supernatants were analyzed for the secretion of recombinant antibodies. The antibody concentration and antibody reactivity were determined by a sandwich enzyme-linked immunosorbent assay (ELISA). Briefly, high-binding-capacity ELISA plates (Corning) that were pre-coated with either goat anti-mouse IgG (Sigma) or egg albumin were incubated for 1 h with 200 μl of DMEM containing 10% FBS for blocking. After washing the plates 3x with PBS, the cell-culture supernatant was transferred into the ELISA plates and incubated for 2 h at room temperature. After washing the plates with PBS, 100 μl of alkaline phosphatase (AP)-conjugated goat anti-mouse IgG (Sigma) at a concentration of 0.8 μg/ml in PBS was added to the wells and incubated for 2 h. After washing with PBS, the assays were developed using BM Chemiluminescence ELISA Substrate (AP) (Roche) and quantified with a Tecan GENios microplate reader (TECAN, Crailsheim, Germany). The magnitude of the light emission was expressed as relative light units (RLU).

## Abbreviations

TS-HR: target-selective homologous recombination; V: immunoglobulin variable; VL: immunoglobulin light chain variable; VH: immunoglobulin heavy chain variable; 5' RACE: rapid amplification of 5' cDNA ends; ELISA: enzyme-linked immunosorbent assay; AP: alkaline phosphatase; IgG: immunoglobulin gamma; RT-PCR: reverse-transcription polymerase chain reaction; RLU: relative light unit; PCR: polymerase chain reaction

## Authors' contributions

NK designed and performed the experiments and drafted the manuscript. MY performed the experiments and analyzed the data described in this study. MI organized the experiments. All authors read and approved the final manuscript.
